# Codelivery of a cytotoxin and photosensitiser *via* a liposomal nanocarrier: a novel strategy for light-triggered cytosolic release†
†Electronic supplementary information (ESI) available. See DOI: 10.1039/c8nr04048f


**DOI:** 10.1039/c8nr04048f

**Published:** 2018-10-18

**Authors:** Elnaz Yaghini, Ruggero Dondi, Karen J. Edler, Marilena Loizidou, Alexander J. MacRobert, Ian M. Eggleston

**Affiliations:** a Division of Surgery and Interventional Science , University College London , Royal Free Campus , Rowland Hill Street , London NW3 2PE , UK . Email: a.macrobert@ucl.ac.uk ; Email: elnaz.yaghini@ucl.ac.uk; b Department of Pharmacy and Pharmacology , University of Bath , Bath BA2 7AY , UK . Email: ie203@bath.ac.uk; c Department of Chemistry , University of Bath , Bath BA2 7AY , UK

## Abstract

Endosomal entrapment is a key issue for the intracellular delivery of many nano-sized biotherapeutics to their cytosolic or nuclear targets. Photochemical internalisation (PCI) is a novel light-based solution that can be used to trigger the endosomal escape of a range of bioactive agents into the cytosol leading to improved efficacy in pre-clinical and clinical studies. PCI typically depends upon the endolysosomal colocalisation of the bioactive agent with a suitable photosensitiser that is administered separately. In this study we demonstrate that both these components may be combined for codelivery *via* a novel multifunctional liposomal nanocarrier, with a corresponding increase in the biological efficacy of the encapsulated agent. As proof of concept, we show here that the cytotoxicity of the 30 kDa protein toxin, saporin, in MC28 fibrosarcoma cells is significantly enhanced when delivered *via* a cell penetrating peptide (CPP)-modified liposome, with the CPP additionally functionalised with a photosensitiser that is targeted to endolysosomal membranes. This innovation opens the way for the efficient delivery of a range of biotherapeutics by the PCI approach, incorporating a clinically proven liposome delivery platform and using bioorthogonal ligation chemistries to append photosensitisers and peptides of choice.

## Abstract 2

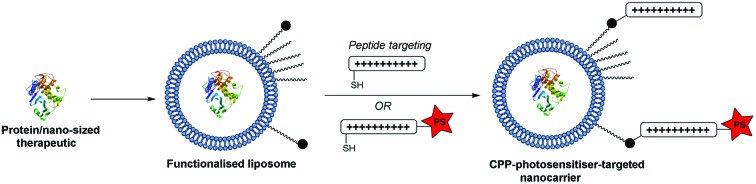
Light-triggered intracellular delivery of a protein toxin was achieved by codelivery *via* a liposomal nanocarrier, targeted with a cell-penetrating peptide (CPP)–photosensitiser conjugate.

## 1. Introduction

The intracellular delivery of many promising biotherapeutics and nanomedicines is often hampered by endosomal entrapment.^1^ This can affect many important protein therapeutics and protein-targeted drugs,^2^ as well as emerging nucleic acid therapeutics developed for gene therapy applications.^3^ Macromolecular therapeutics are typically taken up into the cell by endocytic processes, whereupon they remain sequestered in endo/lysosomes, and are both prevented from reaching their intracellular targets and are susceptible to degradation by lysosomal enzymes.^4^ A number of approaches have been investigated to facilitate the escape of entrapped therapeutics, such as the addition or coadministration of an endosome-disrupting agent with the entrapped material,^5^ however a potentially much more flexible solution is one where escape can be triggered by an external stimulus such as light.^6^,^7^

Photochemical internalisation (PCI)^8^,^9^ is a highly effective approach for effecting endosomal release that relies upon the light-based technique of photodynamic therapy (PDT).^10^ In PCI, endosomal escape of the therapeutic agent may be triggered with light after coadministration of a suitable photosensitiser alongside the therapeutic agent. Provided that the photosensitiser has the appropriate physical properties it may localise in the endo/lysosomal membranes of the organelles where the therapeutic agent is sequestered, and in this way, upon irradiation the reactive oxygen species (ROS) that are generated induce selective damage and partial rupture of the organelles.^11^,^12^ This allows entrapped molecules to escape to reach their intracellular targets, but the viability of the cells themselves is not compromised. The feasibility and safety of PCI has recently been demonstrated in a clinical context for the treatment of head and neck cancer using bleomycin, a hydrophilic glycopeptide agent that is taken up into cells by endocytosis and normally remains entrapped in endosomes.^13^ Preclinical studies with various therapeutic proteins and protein toxins have also shown similar promise.^14^ A further advantage of PCI compared to other methods of targeted drug release is that it is both spatially and temporally selective, which therefore also provides scope for novel applications in biomedical research such as light-triggered gene silencing^15^ and immunotherapy.^16^

Although a wide range of photosensitisers have been developed for PDT,^17^ only a limited sub-set actually possess the correct properties for use in PCI. The key requirement for a PCI photosensitiser is an amphiphilic character that not only promotes its internalisation *via* endocytosis within the same intracellular vesicle as a coadministered therapeutic, but crucially also allows it to localise specifically in the hydrophobic environment of the endosomal membranes.^18^,^19^ In this way, selective oxidative damage to endosomal membranes can be effected upon irradiation so as to allow the escape of entrapped agents and also the photosensitiser itself. Some of the most effective PCI photosensitisers to date which possess the required intrinsic amphiphilic character are sulfonated tetrapyrrole derivatives, such as the porphyrin, disulfonated tetraphenylporphine (TPPS_2a_), and the disulfonated aluminium phthalocyanine, AlPcS_2_,^18^ as well as the clinically used disulfonated tetraphenyl chlorin derivative, fimaporfin.^20^ We have however shown that the necessary amphiphilic and lysosomotropic properties can be engineered through the conjugation of otherwise highly hydrophobic photosensitisers to cationic cell-penetrating peptides (CPPs), to produce molecules that not only have the desired properties for PCI, but are superior to more classical tetrapyrrole-based derivatives.^21^,^22^ Other researchers have also adopted this principle of peptide-targeting with other photoactivatable dyes,^23^–^26^ and in this context, we recently demonstrated that CPP-conjugation can be a very attractive way of repurposing well-known photosensitisers that are clinically approved for PDT such as chlorin e_6_, which lack the appropriate physical properties for PCI.^27^ This could be easily achieved by the application of biorthogonal ligation chemistry for the attachment of the chosen photosensitiser to a readily available derivatised CPP, opening up the possibility of using other clinical PDT photosensitisers with the most attractive spectroscopic properties (*i.e.* strong absorption in the red region) for PCI purposes.^15^,^17^

Liposome nanocarriers have received much attention for the enhanced delivery of proteins and other biotherapeutics to address issues of toxicity, stability and targeting.^28^–^31^ However, despite considerable investigations into tailoring the lipid composition of these systems,^30^ endosomal entrapment remains a major barrier to effective delivery. An additional issue is that in order for encapsulated agents to exert their effects after internalisation, they must also escape the interior of such nanocarriers,^32^ which ideally might be effected in a directed fashion by application of an external stimulus such as heat or light.^33^,^34^ Fretz *et al*. have demonstrated that the endosomal entrapment of liposome systems may be overcome by PCI using a conventional coadministered photosensitiser,^35^ and such studies have now led to considerable recent interest in the development of light-triggered systems where a photosensitiser is loaded into the liposome structure itself to effect the destabilisation of the bilayer membranes of both the endolysosomes where the nanocarrier is entrapped^15^ and the liposome nanocarrier itself.^36^–^39^ Although this meets the key requirement for PCI of colocalisation of the photosensitiser and entrapped agent, the effectiveness of such systems depends upon the localisation and orientation of a given photosensitiser in the lipid bilayer of the carrier, with incorporation of the photosensitiser itself potentially having a significant effect on the stability of the liposomal formulation.^40^

**Scheme 1:**
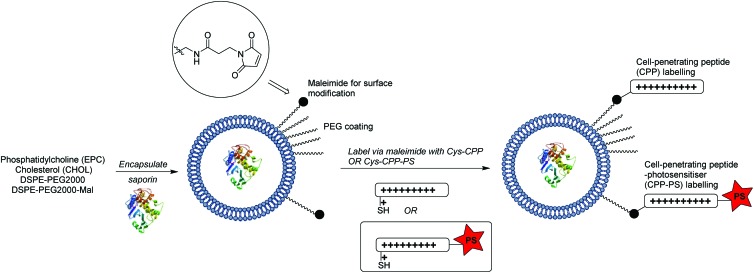
Design and assembly of liposomal nanocarrier presenting an endolysosomally targeted photosensitiser at the surface for light-triggered intracellular release of the liposomal cargo (cytotoxin).

In the present study we have explored whether a more flexible and powerful strategy could be provided by an extension of our CPP-conjugation approach for PCI, as outlined in Scheme 1. The liposomal carrier within which a biomolecule is encapsulated is labelled with CPP units, a certain proportion of which are further functionalised with a hydrophobic porphyrin component connected to the peptide *via* a flexible linker. We reasoned that in this way the photosensitiser would be presented at the exterior surface of the liposome *via* the hydrophilic CPP,^41^ independent of the precise nature of the former, and as such would be able to effectively access endo/lysosomal membranes upon internalisation, providing the required light-triggered escape device for PCI. At the same time, as the photosensitiser is covalently tethered to the nanocarrier, sufficient ROS-mediated damage of the liposome can also be achieved to liberate the biomolecule to be delivered. Further potential advantages of this carrier approach are the possibility to effect the highly controlled delivery of structurally diverse molecules, including ones not normally subject to endosomal entrapment,^33^,^42^,^43^ or where 1 : 1 encapsulation in a suitable “Trojan horse”^44^ is not achievable.

As a proof of concept for such a modular delivery system, we have incorporated a nano-sized protein toxin within such a tailored liposomal system and examined the light-triggered endosomal release of the entrapped cytotoxin in a fibrosarcoma cell line.

## 2. Experimental

### Materials and characterisation

Chemical reagents for peptide synthesis and bioconjugations were obtained from Sigma-Aldrich (Gillingham, UK) and Novabiochem (Nottingham, UK). Peptide grade dimethylformamide (DMF) was obtained from Rathburn Chemicals (Walkerburn, UK). Anhydrous dichloromethane (DCM) was obtained by distillation over calcium hydride. All other solvents were obtained from Fisher Scientific (Loughborough, UK) and used as received. HEPES buffer refers to a solution that was 10 mM HEPES, 137 mM NaCl. DSPE-PEG_2000_ and DSPE-PEG_2000_-maleimide were purchased from Nanocs Inc. (New York, USA). EPC (egg phosphatidylcholine) and cholesterol were obtained from Sigma-Aldrich. Saporin from *Saponaria officinalis* seeds was obtained from Sigma-Aldrich. MC28 cells, a methylcholanthrene-induced rat fibrosarcoma cell line, were grown in DMEM supplemented with 10% FCS at 37 °C in a humidified atmosphere containing 5% CO_2_. Materials for the cell studies were purchased from Sigma-Aldrich. Cellular uptake experiments were performed using a fluorescence microplate reader (Fluoroskan Ascent PL, Thermo Labsystems, Ireland). UV spectra were recorded on a PerkinElmer Lambda 19 uv/vis spectrophotometer. Fluorescence spectra were recorded on a Cary Eclipse fluorimeter. Analytical RP-HPLC was performed on a Dionex Ultimate 3000 system (Dionex, UK), with a VWD-3400 variable wavelength detector. Analyses were performed at 35 ± 0.1 °C on a Gemini 5 μ C18 110 A column, (150 × 4.6 mm – Phenomenex, UK), equipped with a Security Guard C18 (ODS) 4 × 3.0 mm ID guard column (Phenomenex, UK), at a flow rate of 1 mL min^–1^. Mobile phase A was 0.1% aq. TFA, mobile phase B was 0.1% TFA in MeCN. (Gradient: 0.0–10.0 min 0–95% B, 10.0–20.0 min 95% B, 20.0–20.1 min at 95–5% B, 20.1–23.0 min 5% B.) Preparative RP-HPLC was performed on a Dionex HPLC system equipped with a Phenomenex Gemini 5 μ C18 (250 × 10 w mm) column at a flow rate of 2.5 mL min^–1^. High resolution mass spectrometry was performed using a Bruker MicroTOF autospec ESI mass spectrometer.

### Preparation of peptide derivatives

#### H-Gly-Lys-Lys-Arg-Arg-Gln-Arg-Arg-Arg-Gly-Tyr-Lys-Cys-NH_2_ (**1**) (*Cys-CPP*)

The unlabelled Tat(48–57) derivative was synthesised by Fmoc strategy as described previously.^45^

#### 11-Azido-undecanoyl-Gly-Lys-Lys-Arg-Arg-Gln-Arg-Arg-Arg-Gly-Tyr-Lys-Cys(S*t*Bu)-NH_2_ (**2**)

The synthesis of the ligatable Tat(48–57) derivative was performed on 250 mg of resin (0.15 mmol scale) by Fmoc strategy on Rink Amide MBHA Resin (Novabiochem, 200–400 mesh, 0.60 mmol g^–1^ loading). Removal of the Fmoc group from the resin was performed manually at room temperature with 20% piperidine/DMF (2.5 mL, 4 × 3 min), using a disposable plastic reactor (Grace UK). Attachment of the first residue was performed according to the method of Han *et al*., using a 1 h coupling with no preactivation and Fmoc-Cys(SS*t*Bu)-OH (4 eq.), HATU (4 eq.), HOBt (4 eq.) and collidine (4 eq.). This was followed by an acetylation step (Ac_2_O/DIPEA/DMF = 1/1/8, 2.5 mL, 1 × 10 min). The rest of the peptide sequence was assembled on an Activo P11 automated synthesiser, with subsequent Fmoc deprotection steps being performed using 25% piperidine/DMF (3 mL, 1 × 5 min, 1 × 10 min), and the chain elongation steps being performed at 60 °C for 35 min using 3 eq. of each Fmoc-protected amino acid (Fmoc-Arg(Pmc)-OH, Fmoc-Gln(Trt)-OH, Fmoc-Lys(Boc)-OH, Fmoc-Tyr(*t*Bu)-OH or Fmoc-Gly-OH), PyBOP (3 eq.) and DIPEA (6 eq.) in DMF (4 mL). Acylation of the N-terminus was performed manually with 11-azido-undecanoic acid (4 eq.) and a 5 min preactivation using HATU (4 eq.), HOBt (4 eq.), and DIPEA (6 eq.) in DMF (4 mL). The peptide resin was washed thoroughly with DMF and DCM, then it was treated with TFA/TIS/H_2_O (95/2.5/2.5) for 3 h. The resin beads were filtered off and the filtrate was evaporated to a small volume and then added dropwise to cold anhydrous Et_2_O. The precipitated material was collected by centrifugation, washed twice with Et_2_O, dissolved in 1% aq. TFA, filtered using a 0.2 μm syringe filter and the resulting solution was purified by semi-preparative HPLC (see General Remarks). The purified peptide was then freeze-dried to give **2** (52.4 mg, 11%) as a white powder. *R*_t_ HPLC: *t*_R_: 5.88 min; ESI-HRMS^+^: calcd for C_79_H_150_N_37_O_16_S_2_: 645.7162, found: 645.7169 [M + 3H]^3+^.

#### Porphyrin–peptide conjugate (**4**)

A solution of peptide **1** (25.9 mg, 8.17 μmol) and porphyrin derivative **3**^22^ (18.0 mg, 19.0 μmol) in DMSO (2 mL) was stirred at room temperature overnight, shielded from light. The mixture was diluted with 1.0% aq. TFA and directly purified by semi-preparative HPLC. This gave **4** as a dark green freeze-dried solid (21.2 mg, 63%). HPLC *t*_R_: 7.86 min; UV-vis (0.1% aq TFA), nm: 436, 521, 557, 596, 654; ESI-HRMS^+^: calcd for C_155_H_214_N_46_O_19_S_2_ 1030.2284; found: 1030.2397 [M + 3H]^3+^.

#### Porphyrin–peptide conjugate (**5**) (*Cys-CPP-PS*)

A nitrogen-degassed solution of **4** (6.0 mg, 1.5 μmol) in 0.1 M aq NH_4_HCO_3_ buffer (1 mL) was treated with a degassed solution of DTT (4.6 mg, 30.0 μmol) 0.1 M aq NH_4_HCO_3_ buffer (100 μL). The mixture was stirred for 2 h at room temperature, shielded from light, then it was directly injected into semi-preparative HPLC for purification. This gave **5** (5.8 mg, 98%) as a dark-green freeze-dried powder. *R*_t_ HPLC: *t*_R_: 7.88 min; ESI-HRMS^+^: calcd for C_151_H_210_N_46_O_19_S: 750.9145, found: 750.9192 [M + 4H]^4+^.

### Alexa fluor-labelled saporin

Saporin was reconstituted using 10 mM HEPES buffer (2 mL, 135 mM NaCl, pH 7.4) and desalted using Amicon Ultra (Ultra-15, MWCO 10 kDa) spin filters. For a typical labelling, 200 μL of saporin (5 mg mL^–1^ in HEPES buffer) was treated with a solution of Alexa Fluor 488 isothiocyanate in DMSO (20 mM, 25 μL) and gently mixed for 1 h while shielded from light. Excess dye was removed by spin filtering and the labelled saporin was diluted to 1 mL for a final concentration of 1 mg mL^–1^.

### Preparation and characterisation of liposomes

#### Preparation of liposomes

For a typical preparation, EtOH stock solutions containing EPC (14.2 mg, 16.7 μmol), cholesterol (7.7 mg, 20.0 μmol), DSPE-PEG_2000_ (0.69 mg, 0.25 μmol), and DSPE-PEG_2000_-maleimide (0.46 mg, 0.16 μmol) respectively were combined and the mixture was evaporated first with a rotavapor, then under high-vacuum to form a lipid film. The film was then hydrated using HEPES buffer (1 mL, or 1 mL of 1 mg mL^–1^ saporin in HEPES buffer for saporin-loaded liposomes), heated at 60° C for 10 min and vortexed to form the liposomes. The liposomes were then sized 41 times using 200 nm polycarbonate filters at 70° C in an Avanti Polar Lipids miniextruder. The liposomes were purified to remove non-encapsulated saporin by chromatography on Sepharose CL-4B, and eluted with HEPES buffer.

#### Surface conjugation

A purified suspension of liposomes (1 mL) was treated at 4° C with a solution of peptide **1** (0.8 mg, 0.21 μmol) in DMSO (29.6 μmol) and a solution of porphyrin–peptide conjugate **5** (0.2 mg, 0.05 μmol) in DMSO (40 μL). The liposomes were then incubated at 4° C for 18 h and purified to remove excess unconjugated peptides by chromatography on Sepharose CL-4B and eluted with HEPES buffer.

#### Characterisation

Liposome size was characterised by Dynamic Light Scattering using a Malvern Zetasizer Nano. Liposome preparations were sampled twice and each sample as measured in triplicate and the results were averaged. Only one peak was observed in both intensity and volume distributions. Saporin-loaded TPP–Tat liposomes were determined to have a diameter = 180 nm and polydispersity index 0.161. The concentrations of photosensitiser and saporin were determined by analogy with the method of Sardan *et al*.^46^ and Oh *et al*.^47^ (see ESI†).

### Cellular uptake studies

MC28 cells were seeded in 96-well plates overnight with 5000 cells per well. The cells were then incubated with the porphyrin–peptide-conjugated liposomes without encapsulated saporin (empty liposomes) and liposomes containing saporin for 24 h or 48 h. After incubation, the cell medium was removed, cells were washed with PBS and PBS (100 μL) was added into each well. The intracellular levels of the peptide–porphyrin conjugate following uptake of the liposomes were assessed using the fluorescence of the porphyrin following excitation at 405 nm and fluorescence was detected at 650 nm. All experiments were performed in triplicate.

### *In vitro* PDT and PCI

MC28 cells were seeded in 96-well plates for 24 h with 3000 cells per well. The medium was then removed and replaced with fresh medium containing increasing concentrations of empty liposomes or saporin-containing liposomes for 24 h. For PDT (empty liposomes), the concentrations used were 25, 50 and 100 nM. For the PCI combination therapy (saporin-containing liposomes), the respective concentrations of porphyrin and saporin (in parentheses) used in nM were 25 (2.8), 50 (5.8) and 100 (11.4). Control studies were also carried out using liposomes containing saporin, but with surface conjugation with peptide **1** alone, *i.e.* no attached porphyrin moiety. In all cases, following incubation with the liposome samples, the cell medium was removed, the cells were washed thoroughly with PBS, then fresh full medium without the liposomes was added and the cells were finally incubated for 4 h prior to illumination. Illumination was carried out for up to 5 min using a blue LumiSource® flatbed lamp with peak emission at 420 nm and 7 mW cm^–2^ output (PCI Biotech, Oslo, Norway). Cell viability was evaluated at either 48 or 96 h after light illumination using the standard MTT (3-(4,5-dimethylthiazol-2-yl)-2,5-diphenyltetrazolium bromide) assay. All experiments were performed in triplicate. Control groups with no drugs added and with or without light were also assessed.

### Subcellular localisation

For confocal microscopy MC28 cells were seeded in cover glass-bottomed dishes (Fluorodish, World Precision Inst. UK), at a density of 4000 cells per dish for 24 h. The cells were then incubated with the saporin-containing liposomes (2 μM porphyrin concentration) for 24 h. For these imaging experiments, the saporin was labelled with Alexa Fluor 488 as described above. The cell medium was removed, and the cells were then washed with PBS, resuspended with fresh phenol red-free medium, and imaged using an Olympus Laser scanning confocal microscope (FluoView FV1000, 60× magnification, NA 1.20, Olympus UK Ltd, Essex, UK). Fluorescence from the porphyrin was recorded within the range of 620–720 nm using a 405 nm laser for the excitation. Control images were recorded after 24 h incubation with empty TPP-Tat liposomes at the same porphyrin concentration to confirm the absence of any autofluorescence contribution in the Alexa Fluor detection channel since the porphyrin also absorbs at 488 nm. For Alexa Fluor 488 fluorescence imaging, cells were illuminated at 488 nm and the fluorescence signal was recorded at 510–560 nm. Image analysis and processing of the 16-bit images were performed with ImageJ software.

### Light-induced intracellular redistribution

MC28 cells were seeded in cover glass-bottomed dishes (Fluorodish, World Precision Inst. UK), at a density of 4000 cells per dish for 24 h. The cells were then incubated with Alexa Fluor-labelled saporin-containing liposomes (2 μM porphyrin concentration) for 24 h, and on-stage illumination at 405 nm was performed. An intial image of the Alexa Fluor fluorescence was recorded as described above and then further images were recorded at set delays up to 2 minutes following on-stage illumination for 60 s at 405 nm, where the porphyrin absorbs strongly, in order to induce redistribution of the labelled saporin.

### Statistical analysis

Data were analysed using two-tailed Student's *T*-test with appropriate *post hoc* testing using Prism 6 software. Error bars from the mean show ± standard deviation (SD). Values of *P* < 0.05 were considered to be statistically significant. Synergistic cell-killing upon PCI was assessed using the following equation to calculate the value of alpha (*α*):
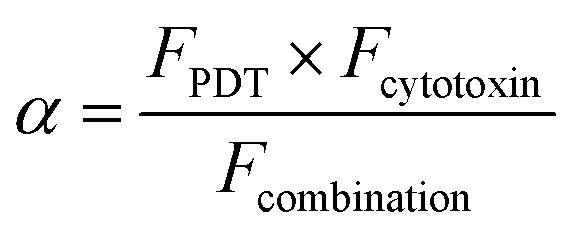
where *F* is the fractional viability for each separate therapy (*i.e.* PDT and the application of the cytotoxin), and the denominator is the fractional viability observed following the PCI combination treatment. If *α* > 1 then a synergistic effect is observed; *α* < 1 denotes an antagonistic effect.^48^

## 3. Results and discussion

### Synthesis of targeted liposomes

The coupling of cell penetrating peptides such as Tat (48–57) to the outer surface of liposome particles is a well-established principle for enhancing the translocation of such drug-carrying materials across cell membranes.^35^,^49^–^52^ Building on our previous studies with CPP-conjugated photosensitisers,^21^,^22^ the novel aspect of our nanocarrier approach for PCI (see Scheme 1) is that the photosensitiser moiety that provides an endosomal escape trigger is covalently linked to the carrier in a highly predictable fashion *via* such a CPP. This ensures that the photosensitiser can intercalate effectively into the lipid bilayer of endosomal membranes to effect oxidative damage and membrane disruption upon irradiation. Moreover, the design provides a simple means to control the photosensitiser loading and distribution over the carrier surface.

Liposomes were prepared by solvent evaporation/hydration^52^ using egg-derived phosphatidylcholine (EPC) with 1.1 mol% PEG_2000_DSPE (0.4 mol% of PEG chains terminated with a maleimide group). They were then surface functionalised *via* thiol-maleimide ligation^53^,^54^ with peptide **1**, a C-terminally extended version of the Tat (48–57) peptide sequence described by Santra *et al.*,^55^ and the related peptide **5** which was additionally labelled at the N-terminus with a tetraphenylporphyrin photosensitiser. Liposomes prepared as above were also loaded with the 30 kDa ribosome-inactivating protein, saporin, tagged with an independent fluorescent label (Alexafluor 488). Saporin and related conjugates has been extensively studied in a range of PCI investigations, both *in vitro* and *in vivo*. It is taken up by endocytosis and extensively trapped in endosomes, which thus provides an excellent model for the delivery of poorly absorbed nano-sized therapeutics that may benefit from PCI technology.^9^

**Scheme 2:**
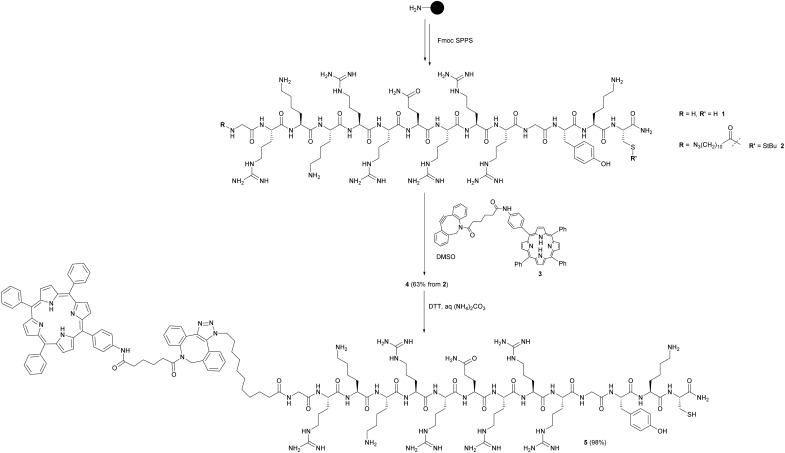
Preparation of targeting peptides **1** and **5**. The unlabelled Tat peptide **1** corresponds to the “*Cys-CPP*” unit in Scheme 1, while peptide **5** which is labelled at the N-terminus with a porphyrin photosensitiser corresponds to the “*Cys-CPP-PS*” unit.

As outlined in Scheme 1, the maleimide-functionalised DSPE component of the liposomes provides a thiol-reactive handle for the attachment of the Cys-containing Tat peptide units **1** and **4**. The preparation of the targeting peptides **1** and **4** is shown in Scheme 2. Peptide **1** (*Cys-CPP*) was synthesised as described previously by us^45^ by Fmoc solid phase peptide synthesis on Rink amide MBHA resin, with trityl protection on the Cys side chain, and with cleavage from the resin and global deprotection being effected with TFA/TIPS/EDT. For peptide–porphyrin conjugate **5** (*Cys-CPP-PS*) the peptide sequence was first assembled with incorporation of the cysteine residue as Cys(S*t*Bu), and the N-terminus was acylated on resin with 11-azidoundecanoic acid. Cleavage from the resin with TFA/TIPS/H_2_O provided the intermediate azidopeptide **2** which was then combined with the ligatable tetraphenylporphyrin derivative **3**^22^ (2 eq.) *via* strain-promoted azide–alkyne cycloaddition (SPAAC)^56^ to give the bifunctional conjugate **4** in 63% yield following HPLC isolation (Scheme 2). Finally, the Cys residue of **4** was selectively unmasked by reduction using DTT to give **5** which was able to undergo a second bioconjugation with the maleimide-bearing liposome surface.

The final targeted nanocarriers were characterised by DLS and UV/VIS absorbance and fluorescence spectroscopy. No significant perturbation of the porphyrin emission spectrum was observed for the labelled liposomes (see ESI†), consistent with the presentation of the photosensitiser unit on the exterior of the nanocarrier, rather than embedded in the lipid phase.^53^ Consistent with this observation, for saporin-loaded TPP–Tat liposomes with diameter 180 nm, the number of pendant porphyrins per liposome can be estimated to be *ca.* 250, with an average linear separation of 20 nm,^57^ such that any neighbour-to-neighbour self-quenching interactions should be negligible.^58^

### Cellular uptake and localisation

**Fig. 1:**
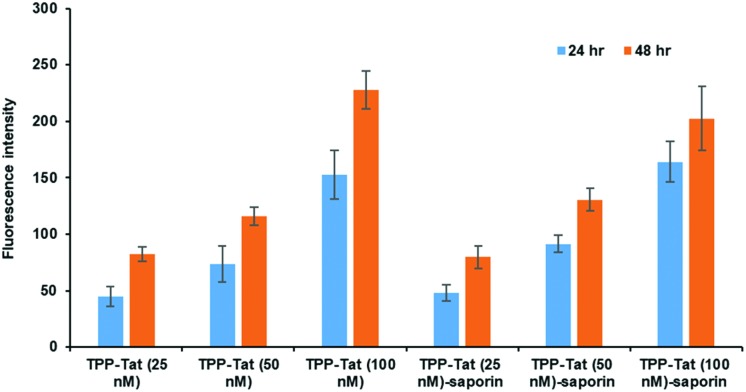
*In vitro* cellular porphyrin fluorescence levels of liposome conjugates in MC28 cells. Cells were incubated with TPP–Tat liposome conjugates or TPP–Tat–saporin liposome conjugates at various concentrations (25, 50, and 100 nM) for 24 h and 48 h. Porphyrin fluorescence was recorded at 650 nm using excitation at 410 and values in arbitrary units (a.u.) are shown after subtraction of baseline levels measured in control cells without addition of liposomes.

The relative uptake of the peptide-conjugated liposomes with and without encapsulated saporin was examined in MC28 rat fibrosarcoma cells by measuring the cellular fluorescence of the surface-attached porphyrin photosensitiser, as shown in Fig. 1. Two incubation times were compared, 24 and 48 h, and three different doses.

The porphyrin fluorescence increased *versus* the dose for both incubation times and no significant differences were observed in porphyrin cellular fluorescence values between TPP–Tat liposomes with or without saporin. We could therefore conclude that the PDT efficacy of each preparation should be comparable without taking saporin cytotoxicity into account. At porphyrin concentrations of 50 or 100 nM, the fluorescence levels observed after 48 h incubation were only marginally higher than those at 24 h, hence the shorter incubation time was adopted for subsequent PDT/PCI studies.

As noted above, the modification of liposome carriers with CPP has been widely used to enhance their uptake across cell membranes, and where Arg-rich CPP such as Tat(48–57) are employed this typically results in uptake of the nanocarrier *via* endocytic processes and subsequent entrapment in endosomes.^59^,^60^ In this context, Fretz *et al.*^50^ have previously demonstrated that Tat-modified liposomes are taken up *via* endocytosis, with the localisation in endosomal compartments being revealed by fluorescent labelling of one of the lipid components.

**Fig. 2:**
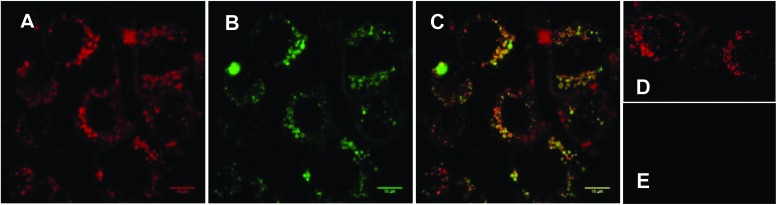
Cellular uptake of TPP–Tat–Saporin–Alexa Fluor 488 liposomal conjugate in MC28 cells using laser scanning confocal microscopy. Cells were incubated with the conjugate for 24 h. Red colour in (A) represents the porphyrin fluorescence signal from TPP–Tat (excitation: 405 nm). Green colour in (B) represents the fluorescence signal from Saporin–Alexa Fluor 488 (excitation at 488 nm). C: Merged image of A and B; D: porphyrin fluorescence from empty TPP–Tat liposomes (excitation at 405 nm). E: control image of same field as D with empty TPP–Tat liposomes (excitation at 488 nm and detection at 510–560 nm). All images on same scale; scale bar: 10 μm.

In order to verify that our nanocarrier system provided the desired subcellular colocalisation of saporin and the associated photosensitiser upon internalisation, and before irradiation, we compared the intracellular distribution of the porphyrin moiety and encapsulated Alexa Fluor-labelled saporin using confocal fluorescence microscopy. Fig. 2A and B show that there is a punctate subcellular localisation for both the porphyrin (red) and saporin (green) fluorescence respectively, which is coincident when merged in Fig. 2C, thus confirming that our strategy should provide the optimum physical colocalization of photosensitiser and bioactive agent in the same subcellular compartment required for effective PCI. Additional control images, 2D and E, recorded after incubation with empty TPP–Tat liposomes confirm the presence of porphyrin signal but the absence of any autofluorescence contribution in the Alexa Fluor detection channel.

**Fig. 3:**
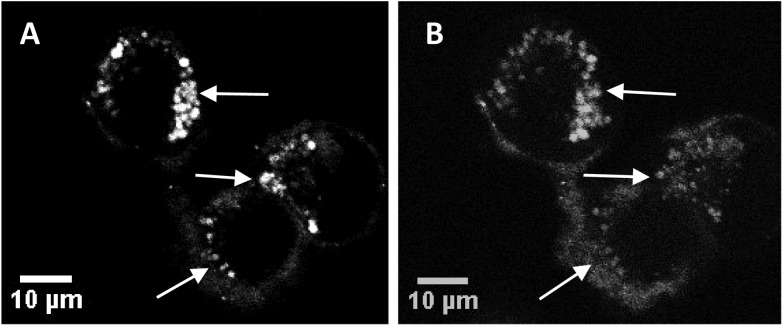
Cellular uptake and photo-induced redistribution of saporin–Alexa Fluor 488 liposomal conjugate in MC28 cells (A) before light illumination, (B) after light illumination using 405 nm laser for the excitation. Middle arrow highlights an area where the Alexa Fluor fluorescence is initially punctate (A) and transitions to a more diffuse distribution (B); top and bottom arrows highlight bright spots of fluorescence that are absent after illumination due to dilution of the fluorescence. Each figure is from the same field and confocal plane. Scale bar: 10 μm. Light illumination on-stage was effected for 60 s and the image was recorded after a further 60 s delay.

Fig. 3A and B show sequential images of the effect of prolonged on-stage illumination using the confocal microscope 405 nm laser on the intracellular fluorescence of the liposome-encapsulated saporin labelled with Alexa Fluor. Acquisition of Fig. 3A used an exposure time of only 1 s, which did not result in detectable perturbation of the nascent fluorescence distribution. However, after a further 60 s of on-stage exposure to the laser a repeat image scan (with a 1 s exposure), recorded 60 s after the 405 nm illumination had ended, showed that the initial punctate intracellular fluorescence had partly dispersed and was consequently weaker (Fig. 3B). This dispersal of fluorescence is consistent with the PCI mechanism whereby the membranes of the endo/lysosomes harbouring the labelled saporin are disrupted following illumination, allowing the biomolecule to redistribute freely. If the endo/lysosome is ruptured then the maxima of the fluorescence intensities in an area containing these vesicles will decline since the dye is spread over a larger volume and is no longer localised in a confined site. These images also correspond well to those previously obtained by us using a very similar illumination protocol using a Tat–peptide–porphyrin conjugate that was administered separately from fluorescently-labelled saporin.^21^

### Phototoxicity studies

**Fig. 4:**
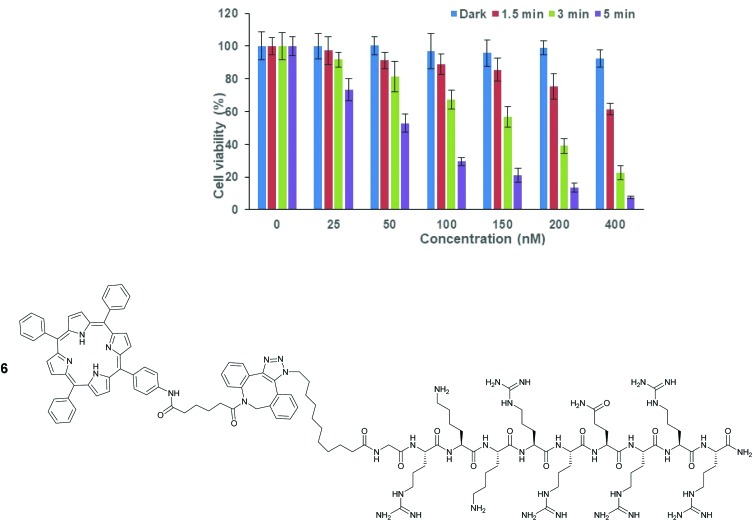
PDT effect of TPP–Tat–peptide liposome conjugation in MC28 cells after incubation for 24 h. Cells were incubated with the nanocarrier at various concentrations and were illuminated either for 1.5, 3 or 5 min. MTT assay was carried out 48 h after light exposure. Data are presented as mean value ± standard deviation (SD). The TPP–Tat–peptide unit conjugated to the liposome surface is directly related to Tat–peptide–porphyrin derivative **6**, previously studied as a stand-alone photosensitiser for PCI.^22^

The phototoxicity of the porphyrin–peptide-conjugated liposomes *without encapsulated saporin*, was examined in MC28 cells following 24 h incubation, as shown in Fig. 4, as a function of concentration and illumination time. The dose–response was assessed in terms of the total porphyrin photosensitiser concentration (present in the solution) *versus* controls without liposome addition, and dark values (*i.e.* without illumination), with the range of concentrations and illumination times being based on previous studies by us with “stand alone” Tat–peptide porphyin derivatives.^21^,^22^ Significant phototoxicity was observed with the empty liposomes which scaled with higher photosensitiser concentration and light doses, and at the highest concentration of 400 nM, cell viability was significantly reduced to 7.4 ± 0.9% using 5 min illumination, while at 50 nM, the viability was 53 ± 5%. Control experiments in the dark showed that there was negligible dark toxicity at concentrations considerably greater than those employed for the PCI experiments (see below).

We have previously reported the phototoxicity of the Tat–peptide–porphyrin derivative **6**^22^ in MC28 cells using the same blue light source. This conjugate is a direct analogue of the surface functionalisation structure present in the liposomes in this study, with the N-terminus of the Tat (48–57) sequence attached to the same porphyrin moiety *via* an identical aliphatic spacer and triazole linkage. Significantly, in the study with **6**, very similar phototoxicity values were observed at 48 h following illumination compared to those obtained with the liposome system here. This supports our hypothesis that such peptide–photosensitiser units displayed on the exterior surface of a liposomal carrier should be able to deliver an effective membrane-damaging effect and endosomal escape.

### Photochemical Internalisation studies (PCI)

**Fig. 5:**
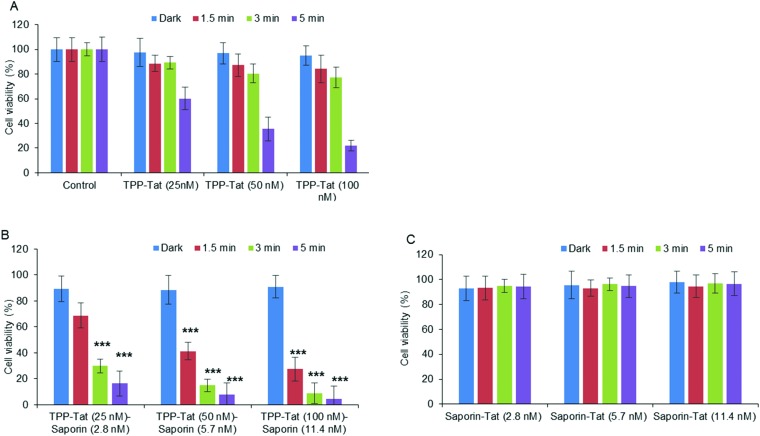
Light-induced cytotoxic response of liposome conjugates in MC28 cells, showing (A) PDT (TPP–Tat liposome conjugates without saporin), (B) PCI (liposome conjugates containing both TPP–Tat and saporin), and (C) Tat conjugates with saporin but without TPP. Cells were incubated with various concentrations of nanocarrier and saporin (TPP–Tat/Tat: 25, 50, and 100 nM, saporin: 2.8, 5.7, and 11.4 nM) and were illuminated for up to 5 minutes. MTT assay was carried out 96 h after light exposure. Data are presented as mean value ± standard deviation (SD) of three independent experiments. *** shows significance *P* < 0.0001 relative to control cells without liposomes.

Following on from the PDT studies, saporin-encapsulated liposomes prepared as above were evaluated in MC28 cells for light-triggered PCI enhancement of saporin cytotoxicity. Saporin has been much used as a model nano-sized agent for PCI studies (diameter: *ca.* 2 nm; MW: *ca.* 30 kDa) as it is prone to entrapment and degradation within lysosomes following endocytosis, thus severely restricting its cytotoxicity when administered directly.^35^ Since PCI is designed to function at *sub-lethal PDT doses*, the optimum porphyrin concentration and light doses were first established using modified liposomes without saporin (empty liposomes). Fig. 5A shows that for porphyrin concentrations at 25–100 nM, a sub-lethal PDT effect with a loss of viability of *ca.* 50% was achieved for all illumination times; for 100 nM the reduction of viability at 5 minutes was larger but still within the range applicable for PCI. It should be noted once more, that at these concentrations, the TPP–Tat liposomes alone elicit no significant toxicity in the absence of light.

Based on these results, we proceeded to test liposomes for PCI with these TPP–Tat concentrations. The same liposomal conjugates with saporin (corresponding concentrations of 2.8, 5.8 and 11.4 nM) elicited only a small reduction in viability of *ca.* 10% without illumination, as shown in Fig. 5B. In contrast, after illumination the viabilities measured for the liposomal conjugates with saporin were significantly reduced. In Fig. 5C, viability measurements are shown for cells incubated with the Tat-conjugated liposomes containing saporin at the same concentrations and the same illumination conditions but without porphyrin conjugation. From Fig. 5B, it can be seen that PCI treatment resulted in significant reductions in viability using 3 or 5 min illumination, *e.g.* using 100 nM TPP and 11.4 nM saporin (*P* < 0.0001). At these concentrations for the loaded nanocarrier, the combined treatment reduced viability significantly to 8.8% at 3 min illumination, compared to 91 ± % viability without illumination. For PDT alone at 100 nM TPP, the viability was 77 ± % (see Fig. 5A). Using the combination of 50 nM TPP and 3 min illumination and 5.7 nM saporin, the overall reduction in viability was lower giving a value of 14.9 ± % but still very significant. Thus, the combined PCI treatment was significantly more effective than the effect of either liposomal saporin or PDT with the empty porphyrin-labelled nanocarrier, consistent with light-triggered release of saporin from endosomes and intracellular relocalisation. Compared to treatment with liposomal saporin alone, PCI reduced the cellular viability by *over an order of magnitude*.

Fig. 5C further emphasises the efficacy of our PCI strategy. Here, the experiments of Fig. 5B were recapitulated using Tat-modified liposomes with no porphyrin labelling. It can be seen that in this case that at all concentrations of the saporin-loaded nanocarrier and all light doses, the cellular toxicity is minimal (*ca.* 95% viability). Thus in the absence of the escape device provided by the porphyrin moiety, the nano-sized toxin remains essentially completely entrapped in endosomes.

The relative synergistic effects achieved *via* light-triggered endosomal escape can be quantified, by the parameter *α*.^48^ This is derived from the fractional cellular viability determined separately upon combined PCI treatment for a given therapeutic, the viability observed for direct administration of the therapeutic alone, and the viability resulting from the PDT effect of a coadministered photosensitiser (see Experimental section). Synergistic effects are indicated by an *α* value significantly greater than unity, whereas if the effect of direct administration and PDT is merely additive, a value of *α* = 1 should be observed. For the results in Fig. 5B, the best effect was achieved with codelivery of 100 nM TPP and 11.4 nM saporin, with an illumination time of 3 min, which gave a value of *α* = 8.0. For comparison, using 50 nM TPP and 5.7 nM saporin for the same illumination time gave a value of *α* = 4.7.

**Fig. 6:**
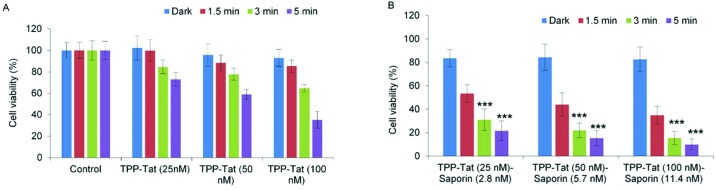
Light-induced cytotoxic response of liposome conjugates in MC28 cells, showing (A) PDT (TPP–Tat liposome conjugates without saporin) and (B) PCI (liposome conjugates conjugates containing both TPP–Tat and saporin) effects. Cells were incubated with various concentrations of TPP–Tat and saporin (TPP–Tat: 25, 50, and 100 nM, saporin: 2.8, 5.7, and 11.4 nM) and were illuminated for up to 5 min. MTT assay was carried out 48 h after light exposure. Data are presented as mean value ± standard deviation (SD) of three independent experiments. *** shows significance *P* < 0.0001.

All other nanocarrier–saporin combinations also delivered PCI synergistic effects on cell viability (see Fig. 6) with the associated combinations of photosensitiser and toxin comparing favourably with those used to achieve similar *in vitro* results when the latter was administered separately with Tat–peptide conjugates bearing the hydrophobic TPP moiety at the N- or C-terminus,^21^,^22^ or a conventional disulfonated tetraphenylporphine derivative.^21^ It is encouraging to note that the *α* values are higher (*i.e.* higher synergicity) using the liposomal coadministration method than obtained in our previous study in the same cell line^22^ where we administered a porphyrin–peptide conjugate analogous to that used here and saporin separately (*α* = 8 *vs*. *α* = 3.5).

When the viability measurements were carried out at 48 h (Fig. 6) after light treatment instead of 96 h, a synergistic PCI effect was also observed, albeit with lower *α* values: codelivery of 100 nM TPP and 11.4 nM saporin with an illumination time of 3 min gave 3.4 for *α* compared to 8 at 96 h. The PCI-induced reduction in viability was also lower at 48 h. The higher efficacy observed at 96 h compared to 48 h reflects the slower onset of the cytotoxic action of released saporin *via* apoptosis and is consistent with our own previous results^61^ and other PCI studies.^62^

## 4. Conclusions

Endosomal entrapment is a major current issue for the effective delivery of nanomedicines. In this study we have demonstrated that cytosolic delivery of a macromolecule that is normally subject to endosomal entrapment can be achieved *via* a liposomal nanocarrier that is functionalised so as to codeliver a membrane-localising photosensitiser for a highly efficient PCI effect. This approach thus simultaneously avoids the practical disadvantage of current PCI protocols whereby the photosensitiser and drug are administered separately, while also enabling effective targeting of the photosensitiser towards the endosomal membrane *via* the peptide component. Unlike other nanocarrier-based approaches, our strategy provides the flexibility to easily vary the loading of the drug to be delivered, while keeping the same level of surface photosensitiser. Moreover, our approach using liposomes as the nanocarrier enables the use of macromolecular drugs as well as small molecule drugs, or even combinations thereof. The modular assembly of the liposomal system also offers scope for the straightforward incorporation of alternative CPPs with inherent tumour-homing properties, as well as other photosensitiser moieties which absorb in the NIR. This should offer a versatile strategy for the targeted and minimally invasive delivery of a range of poorly absorbed therapeutic agents with high potential for clinical translation.

## Conflicts of interest

There are no conflicts to declare.

## Supplementary Material

Supplementary information
