# Supplementary material for: Codelivery of a cytotoxin and photosensitiser via a liposomal nanocarrier: a novel strategy for light-triggered cytosolic release
Source: Nanoscale. 2018 Oct 18;10(43):20366–76. doi: 10.1039/c8nr04048f (PMC6251340; doi:10.1039/c8nr04048f)
Supplement: Supplementary file 1 [file NR-010-C8NR04048F-s001.pdf]

Electronic Supplementary Material (ESI) for Nanoscale.
This journal is © The Royal Society of Chemistry 2018

Electronic Supplementary Material (ESI) for xxxx.
This journal is © The Royal Society of Chemistry 2018

Codelivery of a protein toxin and an endolysosomally targeted photosensitiser via a liposomal nanocarrier: a novel strategy for light-triggered intracellular release of therapeutic macromolecules

Elnaz Yaghini,[a‡*] Ruggero Dondi,[b‡] Karen J. Edler,[c] Marilena Loizidou,[a]
Alexander J. MacRobert[a*] & Ian M. Eggleston[b*]

[a] Division of Surgery and Interventional Science, University College London, Royal Free Campus, Rowland Hill Street, London NW3 2PE, UK

[b] Department of Pharmacy and Pharmacology, University of Bath, Bath BA2 7AY, UK

[c] Department of Chemistry, University of Bath, Bath BA2 7AY, UK

Contents

S2: Calculation of the concentrations of liposomal photosensitiser and encapsulated saporin

S3: HPLC of peptides and peptide-porphyrin conjugates

S5: Mass spectra of peptides and peptide-porphyrin conjugates

S7: UV and Fluorescence spectra

S8: References

This journal is © The Royal Society of Chemistry 2018

Calculation of the concentrations of liposomal photosensitiser and encapsulated saporin

The total number of lipid molecules per liposome in each preparation was estimated using the formula:

$$N_{lipids} = \left[4\Pi\left(\frac{d}{2}\right)^2 + 4\Pi\left(\frac{d}{2} - h\right)^2\right]_{/a}$$

where *d* is the diameter of spherical unilamellar vesicles determined by DLS, *h* is the lipid bilayer thickness and taken as 5 nm (for phosphatidylcholine liposomes[1]), and *a* is the lipid head group area.[2] For unilamellar liposomes composed of phosphatidylcholine the above equation is thus simplified to:

$$N_{lipids} = 17.69\left[\left(\frac{d}{2}\right)^2 + \left(\frac{d}{2} - 5\right)^2\right]$$

with *a* taken as 0.71 nm^2 for phosphatidylcholine.[3]

The number of liposomes per mL was then calculated using the equation:

$$N_{liposomes} = \frac{c \times N_a}{N_{lipids} \times 1000}$$

where *c* is the molar concentration of lipids and N_a is the Avogadro number.

For the calculation of the molarity of the appended TPP moieties within the liposomal nanocarrier, it is assumed that all the maleimido functions on the external layer of the liposome react with a Cys-containing Tat peptide. This is assumed to be half the total number of maleimido units,[4] since by simple distribution, half these units will be displayed on the interior of the liposome. For the available maleimides on the external surface of the liposome, 20% are therefore conjugated with a Tat peptide bearing a TPP unit (based used a 4:1 ratio of peptide **2** (Tat peptide only): **5 (**TPP-Tat peptide). This allows $c_{TPP,}$ the molarity of the TPP moieties, to be calculated as follows:

$$c_{TPP} = \frac{mol_{malemide - PEG2000 - DSPE}}{Volume_{solution}} \times 0.1$$

where $mol_{malemide - PEG2000 - DSPE}$ is the number of moles of maleimide-functionalised lipids used.

For the calculation of the molarity of encapsulated saporin, it is assumed that the internal volume of each liposome has a concentration of 1 mg/mL of saporin, with the molecular weight of labelled saporin taken as 32 kDa. The saporin molecules are confined to a shell (the internal volume of the liposomes)[5] defined by the formula:

$$Volume_{liposome} = \frac{4}{3}\pi\left(\frac{d}{2} - 5\right)^3$$

Using this equation to calculate the internal volume of each liposome, the concentration of encapsulated saporin can be calculated as follows:

$$c_{saporin} = \frac{Volume_{liposome} \times N_{liposomes}}{Volume_{solution} \times N_a}$$

This journal is © The Royal Society of Chemistry 2018

HPLC of peptides and porphyrin-peptide conjugates

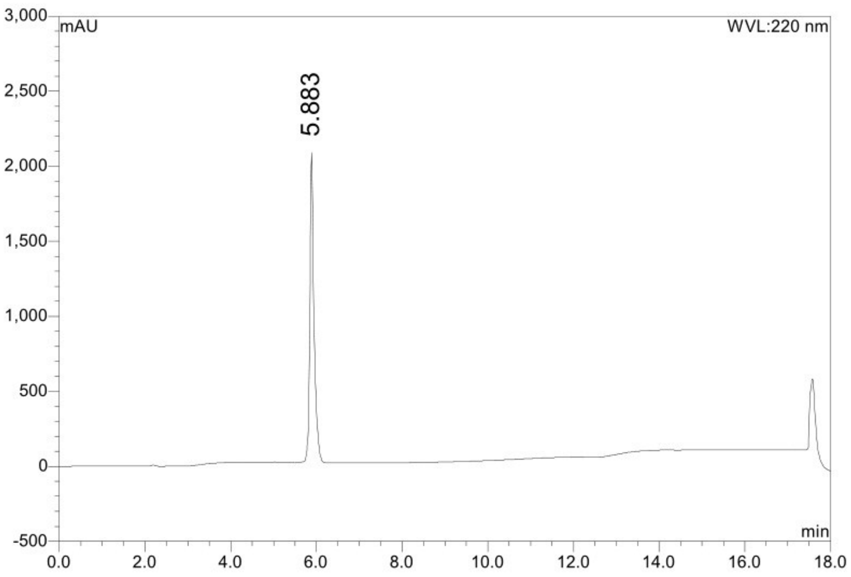

Figure 1 (a). HPLC profile of peptide **2**.

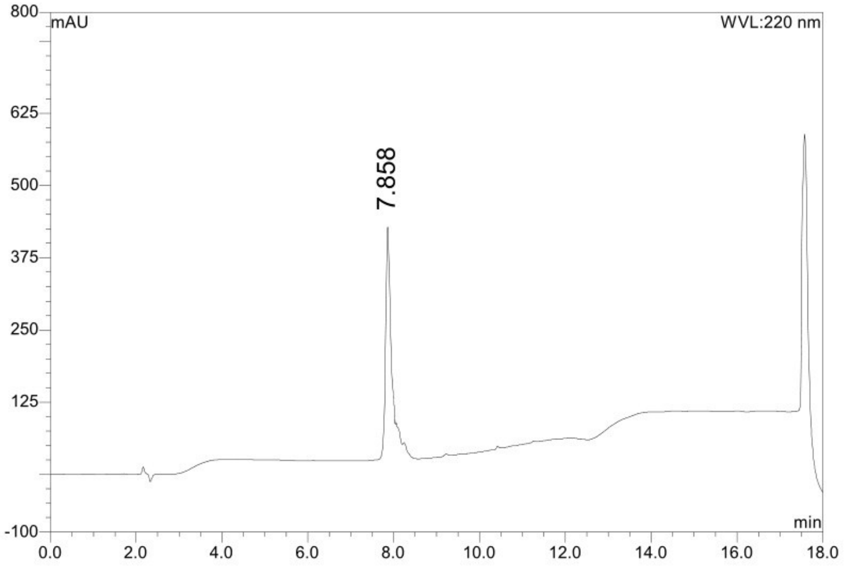

Figure 1 (b). HPLC profile of porphyrin-peptide conjugate **4**.

This journal is © The Royal Society of Chemistry 2018

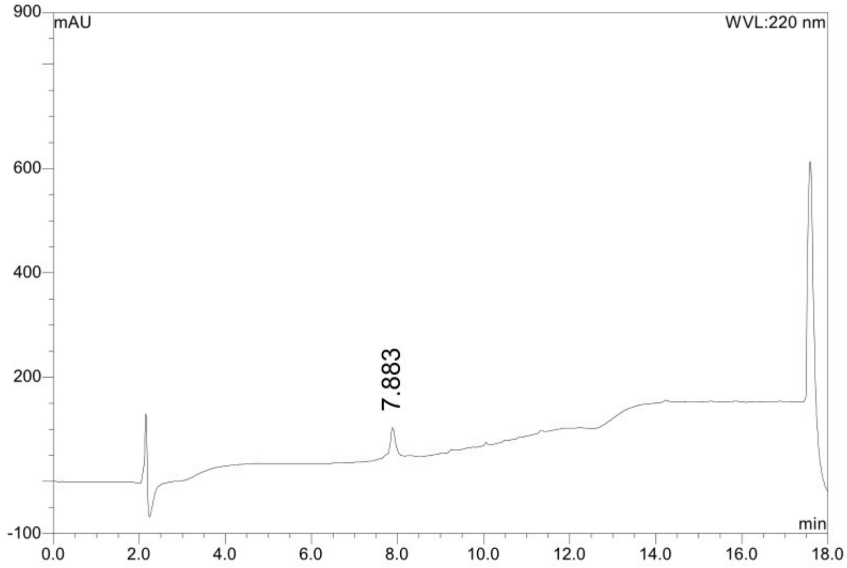

Figure 1 (c). HPLC profile of porphyrin-peptide conjugate **5**.

Mass Spectra of peptides and porphyrin-peptide conjugates

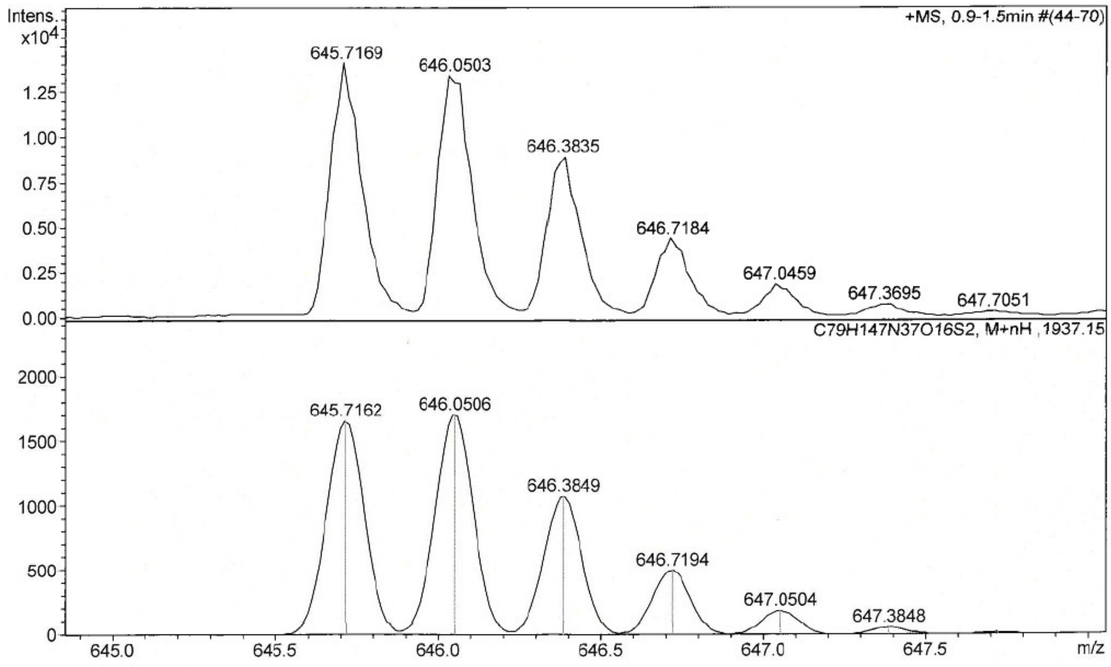

Figure 2 (a). ESI mass spectrum of peptide **2**.

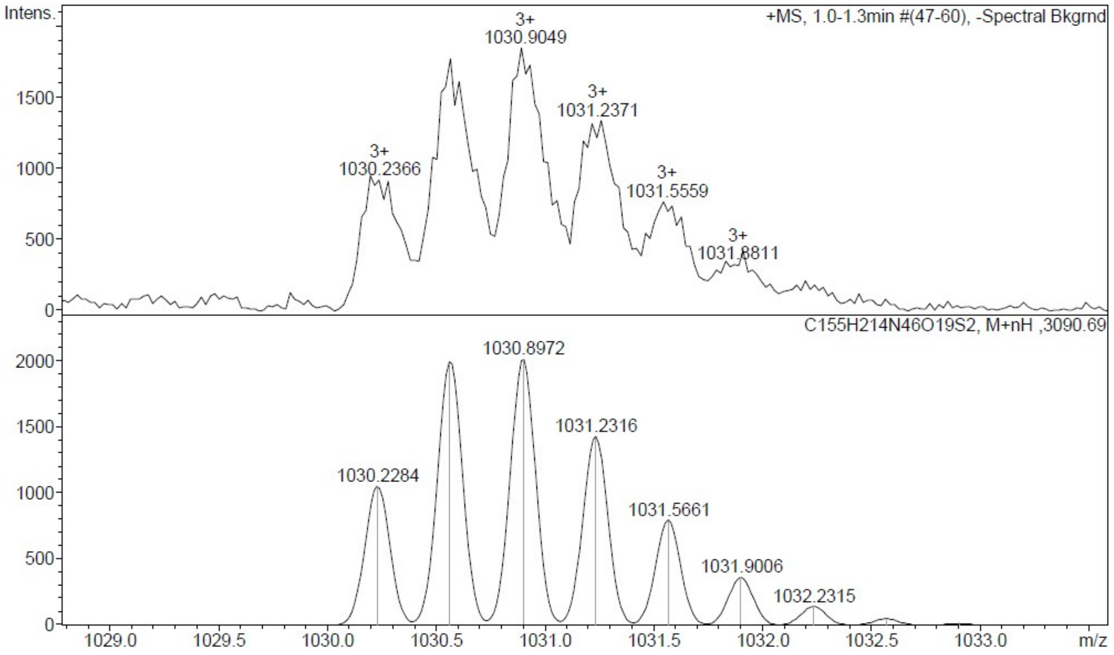

Figure 2 (b). ESI mass spectrum of porphyrin-peptide conjugate **4**.

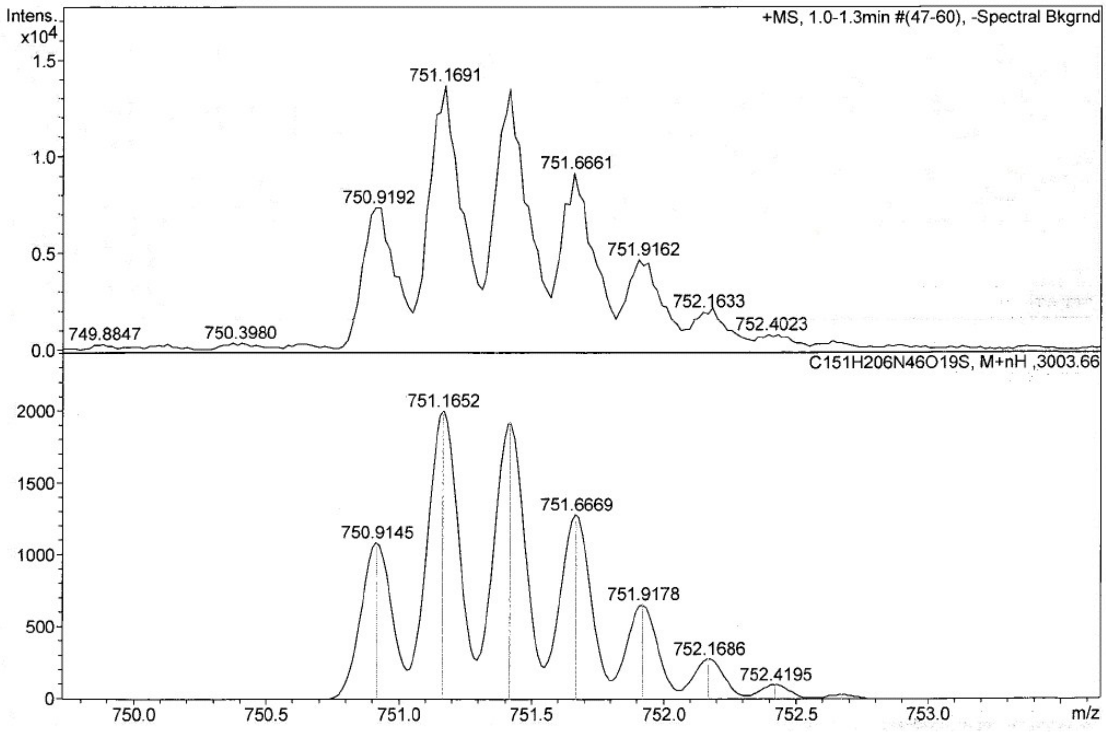

Figure 2 (c). ESI mass spectrum of porphyrin-peptide conjugate **5**.

This journal is © The Royal Society of Chemistry 2018

UV spectra

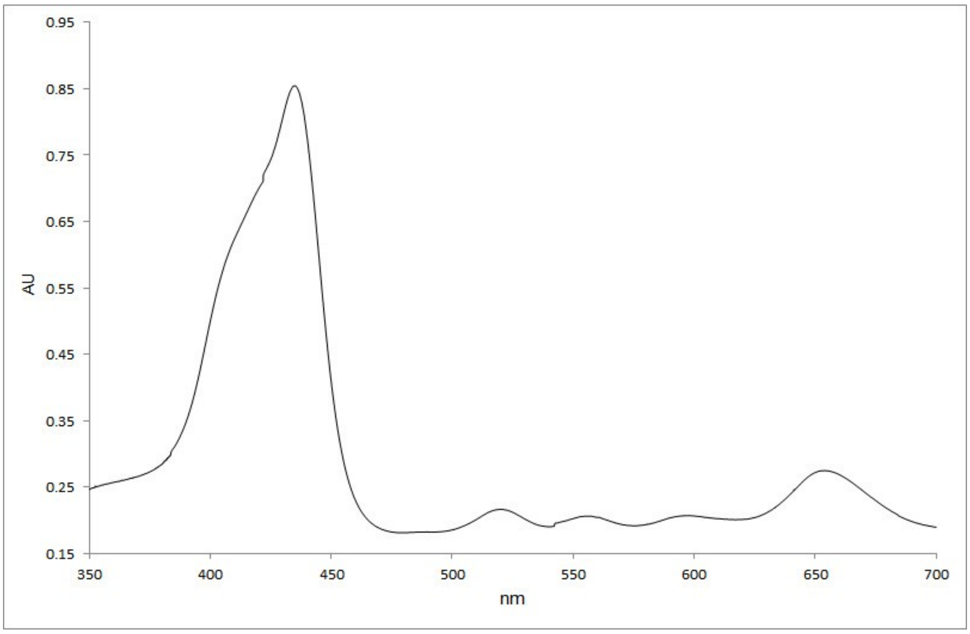

Figure 3 (a). UV-visible spectrum of porphyrin-peptide conjugate **4**.

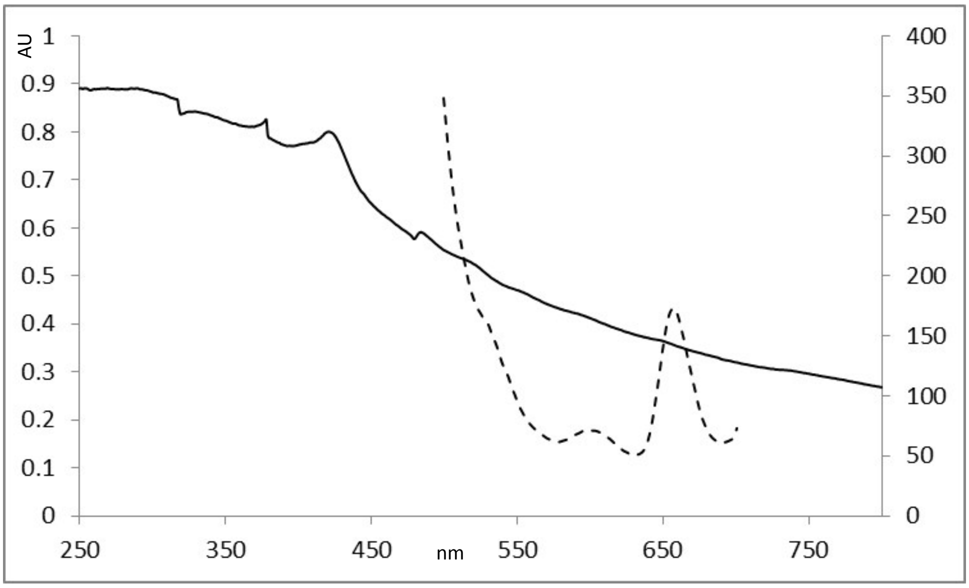

Figure 3 (b). UV-visible (solid line) and fluorescence (dotted line) spectra in HEPES buffer for TPP-Tat liposomes ($\lambda_{exc.}$ = 420 nm).

Electronic Supplementary Material (ESI) for xxxx.
This journal is © The Royal Society of Chemistry 2018

References

1. R. Tero, K. Fukumoto, T. Motegi, M. Yoshida, M. Niwano and A. Hirano-Iwata, *Sci. Rep.* 2017, **7**, 17905.

2. M. Sardan, M. Kilinic, R. Genc, A. B. Tekinay and M. O. Guler, *Faraday Discuss.*, 2013, **166**, 269-283.

3. Encapsula Nanosciences: http://www.liposomes.org/2009/01/number-of-lipid-molecules-per-liposome.html

4. P. Marqués-Gallego and A. I. P. M. de Kroon. *BioMed. Res. Int.* 2014, 129458.

5. K. J. Oh, S. Barbuto, K. Pitter, J. Morash, L. D. Walensky and S. J. Korsmeyer, *J. Biol. Chem.* 2006, **281**, 36999-37008.
